# Enhancing poultry health and productivity through the liver-gut axis with integrated nutritional and immunological approaches: a mini-review

**DOI:** 10.3389/fphys.2025.1537099

**Published:** 2025-02-25

**Authors:** Felix Kwame Amevor, Victoria Anthony Uyanga, Liuting Wu, Dan Xu, Gang Shu, Yingjie Wang, Xiaoling Zhao

**Affiliations:** ^1^ State Key Laboratory of Swine and Poultry Breeding Industry, College of Animal Science and Technology, Sichuan Agricultural University, Chengdu, Sichuan, China; ^2^ Farm Animal Genetic Resources Exploration and Innovation Key Laboratory of Sichuan Province, College of Animal Science and Technology, Sichuan Agricultural University, Chengdu, Sichuan, China; ^3^ Key Laboratory of Livestock and Poultry Multi-omics, Ministry of Agriculture and Rural Affairs, Sichuan Agricultural University, Chengdu, Sichuan, China; ^4^ College of Agriculture, Environmental and Human Sciences (CAEHS) Lincoln University of Missouri, Jefferson City, MO, United States; ^5^ Department of Basic Veterinary Medicine, Sichuan Agricultural University, Chengdu, Sichuan, China

**Keywords:** liver-gut axis, poultry health, nutrition, immunity, *in ovo* stimulation

## Abstract

The liver-gut axis plays a central role in maintaining the health and productivity of poultry. In addition, the liver-gut axis serves as a key regulator of digestion, metabolism, immunity, and detoxification. The gut, with its diverse microbiota, is the primary site for nutrient absorption and immune modulation, while the liver metabolizes nutrients, detoxifies harmful substances, and acts as a frontline defense against pathogens translocated from the gut. Disruptions in this interconnected system, including gut dysbiosis or liver inflammation, can lead to compromised immunity and reduced productivity. This mini-review explores integrated nutritional and immunological strategies aimed at optimizing the liver-gut axis to enhance poultry performance. Nutritional interventions, such as the use of flavonoids, vitamins, amino acids, micronutrients, probiotics, prebiotics, and synbiotics, have demonstrated their potential to support liver and gut health. Dietary components such as phytogenic additives, fiber, and fatty acids further contribute to immune modulation and systemic health. Immunological approaches, such as beta-glucans and *in ovo* stimulation, and molecular approaches, including advanced genetic techniques, offer additional avenues for improving disease resistance and organ function. Despite notable advancements, challenges including antibiotic resistance, environmental stressors, and implementation costs persist. Emerging technologies like metagenomics, metabolomics, and precision breeding offer innovative solutions to enhance liver-gut interactions. This review underscores recent advancements in understanding the liver-gut axis and calls for holistic strategies to improve sustainable poultry production. Future research should integrate these approaches to enhance resilience, productivity, and sustainability in the poultry industry.

## Introduction

The liver-gut axis represents a critical intersection in maintaining the health and productivity of poultry. This dynamic relationship governs key physiological processes such as digestion, metabolism, immunity, and detoxification, all of which are vital for optimal poultry performance. The gut serves as the primary site for nutrient absorption and home to a complex microbiota, while the liver metabolizes these nutrients, detoxifies harmful substances, and coordinates immune responses. The interplay between these organs is essential for sustaining homeostasis and responding to environmental challenges, such as infectious diseases, metabolic stress, and inadequate nutrition (Kogut, 2019; Khan et al., 2020). The gut microbiota has emerged as a pivotal player in the liver-gut axis. It influences not only nutrient availability but also immune modulation and pathogen resistance. Disruptions in the gut microbiota, often due to poor diet or pathogen exposure, can impair liver function, leading to systemic health issues and reduced productivity (Borda-Molina et al., 2018). Similarly, the liver, with its vast network of immune cells, acts as a frontline defense against pathogens that translocate from the gut, which highlights the mutual dependence of these organs in maintaining poultry health (Abd El-Hack et al., 2022).

Integrated approaches targeting the liver-gut axis have gained prominence in the poultry industry. Nutritional strategies such as optimizing macronutrient ratios, supplementing with bioactive compounds like flavonoids, and incorporating probiotics and prebiotics have shown promise in enhancing liver and gut health (Borda-Molina et al., 2016; Ciurescu et al., 2020). Furthermore, immunological interventions, such as the use of synbiotics and *in ovo* stimulation of gut microbiota, further underscore the potential of this axis to improve disease resistance and overall performance in poultry (Siwek et al., 2018). Despite these advances in technology, the liver-gut axis remains a relatively underexplored area in poultry science. There is a growing need for research that elucidates the molecular and immunological mechanisms underpinning this relationship, as well as the development of novel biomarkers and technologies to monitor and optimize liver-gut functions (Talpur et al., 2022). In this mini-review, we explored recent findings on integrated nutritional and immunological approaches to enhancing the liver-gut axis, offering insights into their application for improving poultry health and productivity. By addressing the interplay between diet, immunity, and organ function, this review aims to provide a roadmap for advancing sustainable poultry production practices.

## The liver-gut axis in poultry health

The liver-gut axis is a complex and dynamic network essential for maintaining homeostasis in poultry. Furthermore, the liver-gut axis influences digestion, metabolism, immunity, and detoxification. The gut serves as the primary site for nutrient absorption and is home to a diverse microbial population that plays a critical role in shaping immune responses. These microbiota interact with the host’s immune system, providing protection against pathogens and promoting the development of immune tolerance (Khan et al., 2020). The liver, on the other hand, acts as the central metabolic hub, processing nutrients absorbed by the gut and detoxifying harmful substances. It is also a pivotal immune organ, housing the largest collection of phagocytic cells, including Kupffer cells, which detect and eliminate pathogens translocated from the gut (Anand, and Mande, 2022).

The bidirectional communication between the liver and gut occurs through signaling molecules, microbial metabolites, and immune mediators. Microbial metabolites such as short-chain fatty acids (SCFAs) and bile acids modulate liver metabolism and immunity, while the liver’s bile production shapes the gut microbiota composition, influencing food digestion, nutrient absorption and immune function (Kogut MH. et al., 2018). This interdependence underscores the critical role of the liver-gut axis in maintaining systemic health and promoting poultry productivity.

Disruptions in this axis can have significant consequences for poultry health. Gut dysbiosis, characterized by an imbalance in the microbial community, can lead to increased intestinal permeability and translocation of microbial toxins into the bloodstream, triggering liver inflammation and impairing detoxification and metabolic functions (Borda-Molina et al., 2018; Khan et al., 2020). Similarly, liver inflammation or metabolic overload can negatively impact gut health by altering bile acid composition and reducing the efficiency of nutrient absorption (Negroni et al., 2020). These disruptions often manifest as reduced growth performance, compromised immunity, and increased susceptibility to infectious diseases. For example, a study by Lee and Lillehoj (2021) linked the gut dysbiosis to necrotic enteritis in poultry, a condition that severely impacts intestinal health and liver function (Lee and Lillehoj, 2021). Liver-related conditions such as fatty liver syndrome have also been associated with poor dietary management and microbial imbalances (Habibullah et al., 2024).

Nutritional interventions, such as the inclusion of prebiotics, probiotics, and synbiotics, aim to restore gut microbial balance and enhance immune modulation. Immunological strategies, such as *in ovo* stimulation of the gut microbiota and dietary supplementation with bioactive compounds, have shown potential in reducing liver inflammation and improving overall health (Yitbarek et al., 2019; Ciurescu et al., 2020). Despite the progress in nutritional and immunological strategies targeting the liver-gut axis, significant gaps remain in understanding the molecular mechanisms driving these interactions. Specifically, the lack of detailed knowledge about the signaling pathways, host-microbe interactions, and regulatory networks limits the ability to design precise and effective interventions. Furthermore, reliable biomarkers for early detection of liver-gut dysfunction are not well established, hindering the ability to implement timely corrective measures. Addressing these gaps will not only deepen our understanding of the liver-gut axis but also enable the development of more targeted and sustainable strategies to optimize poultry health and productivity.

## Nutritional strategies to enhance liver-gut health

Optimizing the nutritional profile of poultry diets is a fundamental strategy to enhance the liver-gut axis, thereby improving overall health and productivity. Therefore, targeted dietary interventions with bioactive compounds, essential nutrients, and microbiota-modulating agents are gaining prominence for their multifaceted benefits on gut and liver function (Schwartz and Vetvicka, 2021).

### Flavonoids and vitamins

Flavonoids, naturally occurring polyphenolic compounds, are well-documented for their antioxidant and anti-inflammatory properties. Quercetin, a commonly studied flavonoid, mitigates oxidative stress in hepatocytes and modulates gut microbiota composition, promoting the growth of beneficial microbes such as *Lactobacillus* and *Bifidobacterium* species (Amevor et al., 2022a; Amevor et al., 2022b). In addition, a study reported that vitamin E plays a critical role in maintaining liver and gut health. Vitamin E supports epithelial integrity in the gut, fostering robust mucosal defenses against pathogens, while vitamin E protects hepatocytes from oxidative damage caused by reactive oxygen species (ROS). These vitamins work synergistically to enhance liver-gut homeostasis and boost poultry resilience to environmental stressors (El-Ela et al., 2016; Amevor et al., 2022a; Amevor et al., 2022b).

### Amino acids and micronutrients

Essential amino acids such as methionine play a crucial role in liver detoxification through methylation processes, which are vital for cellular repair and metabolic functions. Methionine also supports the synthesis of glutathione, a potent antioxidant that protects both the liver and gut epithelium from oxidative damage (Bakhshalinejad et al., 2018). Zinc strengthens intestinal tight junctions, reducing permeability and bacterial translocation, while selenium enhances selenoprotein synthesis, which is critical for antioxidant defenses in the liver (Surai et al., 2016). Thus, both nutrients collectively reduce inflammatory responses and oxidative stress, promoting a healthier liver-gut axis (Surai et al., 2016).

### Probiotics, prebiotics, and synbiotics

The modulation of gut microbiota is an emerging focus in poultry nutrition, with probiotics, prebiotics, and synbiotics showing promising results. Probiotics such as *Lactobacillus* and Bifidobacterium species are beneficial microorganisms that improve intestinal health by competing with pathogens and enhancing the production of short-chain fatty acids (SCFAs). SCFAs, particularly butyrate, serve as energy sources for colonocytes and regulate inflammatory responses, indirectly benefiting liver function (Borda-Molina et al., 2016). Prebiotics like inulin act as fermentable substrates for beneficial microbes, further amplifying SCFA production and fostering a balanced microbiota. Synbiotics, a combination of probiotics and prebiotics, offer a comprehensive approach by simultaneously enhancing microbial diversity and gut barrier integrity, thereby improving liver function and reducing systemic inflammation Synbiotics (combination of both probiotics and prebiotics) improve the liver function by enhancing microbial diversity, restoring gut balance, and strengthening the gut barrier. This reduces pathogenic overgrowth, limits toxin leakage, and promotes anti-inflammatory short-chain fatty acid production, and alleviates liver’s detoxification burden. In addition, synbiotics lower systemic inflammation, enhance bile metabolism, and support the liver’s antioxidant capacity, improving nutrient absorption and metabolic efficiency (Markowiak and Śliżewska, 2018).

### Dietary fiber and phytogenic additives

Dietary fiber, an often-underutilized nutrient, supports microbial fermentation in the gut, leading to the production of metabolites such as SCFAs that improve gut integrity and liver metabolic functions (Tejeda and K Kim, 2021). Phytogenic additives, including essential oils and plant extracts like oregano oil and curcumin, have shown antimicrobial and hepatoprotective effects. These compounds reduce gut pathogen load and modulate liver enzyme activity, offering an additional layer of protection to the liver-gut axis (Windisch et al., 2008).

### Fatty acids

Supplementing poultry diets with omega-3 fatty acids, such as those found in fish oil, can have profound effects on the liver-gut axis. Omega-3 fatty acids modulate inflammatory pathways by reducing the production of pro-inflammatory cytokines and promoting the synthesis of anti-inflammatory mediators. This improves liver health and enhances intestinal barrier function, as well as reduce the risk of bacterial translocation and systemic inflammation (Calder, 2015).

### Immunological and molecular interventions

The liver-gut axis in poultry is not only influenced by dietary factors but also by immunological and molecular interventions. Thus, targeting the immune system and genetic pathways offers innovative approaches to enhance health and productivity. These strategies capitalize on the interconnected nature of the gut and liver to bolster disease resistance, improve nutrient utilization, and optimize overall performance in poultry.

#### Immune modulation through nutrients

Nutritional immunomodulators are gaining attention for their role in enhancing the innate immune response in both the liver and gut. Beta-glucans, naturally occurring polysaccharides derived from yeast, fungi, or cereals, are particularly effective in stimulating the immune system. They activate macrophages and dendritic cells, enhancing phagocytosis and cytokine production, which aids in the clearance of pathogens (Cox et al., 2010; Guo et al., 2003). Studies show that dietary beta-glucans significantly reduce mortality rates and improve resistance to infections in poultry, particularly in cases of bacterial and viral challenges (Cox et al., 2010; Guo et al., 2003). Similarly, nucleotides and polyunsaturated fatty acids (PUFAs) have been shown to modulate immune responses by regulating inflammatory pathways and enhancing lymphocyte proliferation (Calder, 2015).

#### *In ovo* stimulation

*In ovo* stimulation, involving the injection of bioactive compounds or probiotics into embryonic eggs, is an innovative technique that has shown promise in promoting liver-gut health. This intervention exposes the developing embryo to beneficial microbes or nutrients, shaping the gut microbiota and immune system before hatching (Dankowiakowska et al., 2019). Several published articles indicate that early colonization of the gut with beneficial microbes, such as *Lactobacillus* or *Enterococcus* species, enhances gut barrier function and reduces the colonization of pathogenic bacteria post-hatch. In addition, *in ovo* stimulation improves the expression of genes related to gut nutrient transporters and immune markers, leading to enhanced nutrient absorption and systemic immunity (Dankowiakowska et al., 2019; Siwek et al., 2018). Moreover, *in ovo* stimulation has been shown to positively influence liver development and function. It can enhance hepatic antioxidant enzyme activity, which protects the liver from oxidative damage during rapid metabolic growth post-hatch (Das et al., 2021). Bioactive compounds delivered in ovo also modulate liver gene expression, promoting better lipid metabolism and reducing fat accumulation in the liver (Duan et al., 2021). In addition, *in ovo* administration of probiotics has been linked to improved hepatic immune responses, helping to mitigate liver inflammation and enhancing overall systemic immunity (Bednarczyk et al., 2016). These effects collectively optimize nutrient utilization and metabolic efficiency, which are essential for poultry health and productivity.

#### Genetic and molecular approaches

The advances in genomics and transcriptomics are revolutionizing poultry health management by identifying molecular pathways that influence the liver-gut axis (Dehau et al., 2022). Key genes associated with liver detoxification, such as those encoding cytochrome P450 enzymes, and gut barrier integrity, including tight junction proteins like occludin and claudin, have been identified as potential targets for genetic selection. Breeding programs now incorporate these insights to develop poultry lines with enhanced disease resistance and metabolic efficiency (Gao et al., 2023).

Moreover, the integration of CRISPR-Cas9 technology offers precise gene editing capabilities, allowing researchers to enhance specific traits such as antimicrobial peptide production or antioxidant enzyme activity (Dehau et al., 2022; Gao et al., 2023). Transcriptomic studies have also highlighted the role of non-coding RNAs, such as microRNAs, in regulating immune responses and liver function. These molecular tools pave the way for precision nutrition, enabling tailored diets that align with genetic predispositions for optimal liver-gut performance (Dehau et al., 2022; Gao et al., 2023).

#### Immunotherapy and vaccination strategies

Immunotherapy and vaccination strategies are critical tools for enhancing immune responses and protecting poultry health. Immunotherapies, including cytokine supplementation, have been widely utilized to boost antiviral and antibacterial defenses in both the gut and liver (Dhama et al., 2015). Cytokines, such as interferons and interleukins, play pivotal roles in modulating immune responses by enhancing pathogen recognition and promoting the activation of immune cells, which are crucial for combating infections (Ruvalcaba-Gómez et al., 2022). For instance, supplementation with interleukin-2 (IL-2) has been shown to improve T-cell activity, while interferon-gamma (IFN-γ) enhances the innate immune response, reducing pathogen replication in the liver and gut (Rothwell et al., 2004).

Vaccines remain indispensable for preventing infections caused by pathogens such as *Salmonella*, *Campylobacter*, and *Escherichia coli*. These vaccines not only reduce the prevalence of infections but also mitigate systemic inflammation, which is vital for maintaining liver health and preventing metabolic disturbances (Pumtang-On et al., 2021). Novel vaccine delivery systems, such as nanoparticle-based vaccines and mucosal vaccines, are being developed to improve efficacy by directly targeting the gut-associated lymphoid tissue (GALT), thereby enhancing localized immune responses and preserving gut barrier integrity (Renu and Renukaradhya, 2020). In addition, the use of recombinant vector vaccines has shown promise in generating robust and long-lasting immunity, while reducing the risk of vaccine-induced adverse effects (Hein et al., 2021).

#### Gut-liver crosstalk and immune tolerance

The gut-liver axis relies heavily on immune tolerance to maintain homeostasis. Dysregulation in this balance can lead to excessive inflammation, compromising both gut integrity and liver function (Tejeda and K Kim, 2021). Emerging interventions aim to modulate regulatory T cells (Tregs) and hepatic Kupffer cells to mitigate inflammation while preserving immune defenses. For instance, dietary interventions that promote SCFA production in the gut, such as fiber supplementation, can enhance Treg activity, reducing liver inflammation and supporting systemic immune health (Tejeda and K Kim, 2021).

### Challenges and future directions

While significant progress has been made, challenges such as antibiotic resistance, high implementation costs, and environmental stressors remain. Emerging technologies like metagenomics and metabolomics offer new avenues for monitoring and optimizing liver-gut functions. In addition, integrated strategies combining nutritional supplements with genetic and immunological approaches hold promise for overcoming these challenges and enhancing poultry health sustainably.

## Conclusion

The liver-gut axis represents a cornerstone of poultry health and productivity. Nutritional and immunological strategies, including the use of probiotics, amino acids, and *in ovo* stimulation, offer practical solutions for optimizing liver-gut functions (Table 1). Future research should focus on leveraging emerging technologies and novel approaches to further enhance the sustainability and profitability of the poultry industry.

**TABLE 1:** Approaches to enhance the liver-gut axis in poultry.

Approach	Description	Effects on liver-gut axis	References
Nutritional interventions	Use of prebiotics, probiotics, synbiotics, flavonoids, vitamins. and animo acids	Improves gut microbiota balance, reduces oxidative stress, and enhances liver detoxification and immunity	Borda-Molina et al. (2018), Schwartz and Vetvicka (2021)
Immunological approaches	Beta-glucans and *in ovo* stimulation of gut microbiota	Enhance immune modulation, reduces systemic inflammation, and improves liver function through antioxidant effects	Das et al. (2021), Bednarczyk et al. (2016)
Genetic and molecular tools	Advance breeding, CRISPR, transcriptomics, and biomarker identification	Improves disease resistance, gut barrier integrity, and metabolic efficiency	Khwatenge and Nahashon (2021), Gao et al. (2023)
Vaccination and immunotherapy	Cytokines, such as interferons, and vaccines targeting specific pathogens	Reduces liver inflammation, enhances systemic immunity, and lowers pathogen burden in the gut and liver	Dhama et al., 2015; Renu and Renukaradhya, 2020
